# Experimental Model Systems for Understanding Human Axonal Injury Responses

**DOI:** 10.3390/ijms22020474

**Published:** 2021-01-06

**Authors:** Bohm Lee, Yongcheol Cho

**Affiliations:** Laboratory of Axon Regeneration & Degeneration, Department of Life Sciences, Korea University, Anam-ro 145, Seongbuk-gu, Seoul 02841, Korea; wiosna328@korea.ac.kr

**Keywords:** neurodegeneration, axonal regeneration, animal models

## Abstract

Neurons are structurally unique and have dendrites and axons that are vulnerable to injury. Some neurons in the peripheral nervous system (PNS) can regenerate their axons after injuries. However, most neurons in the central nervous system (CNS) fail to do so, resulting in irreversible neurological disorders. To understand the mechanisms of axon regeneration, various experimental models have been utilized in vivo and in vitro. Here, we collate the key experimental models that revealed the important mechanisms regulating axon regeneration and degeneration in different systems. We also discuss the advantages of experimenting with the rodent model, considering the application of these findings in understanding human diseases and for developing therapeutic methods.

## 1. Introduction

**Figure 1:**
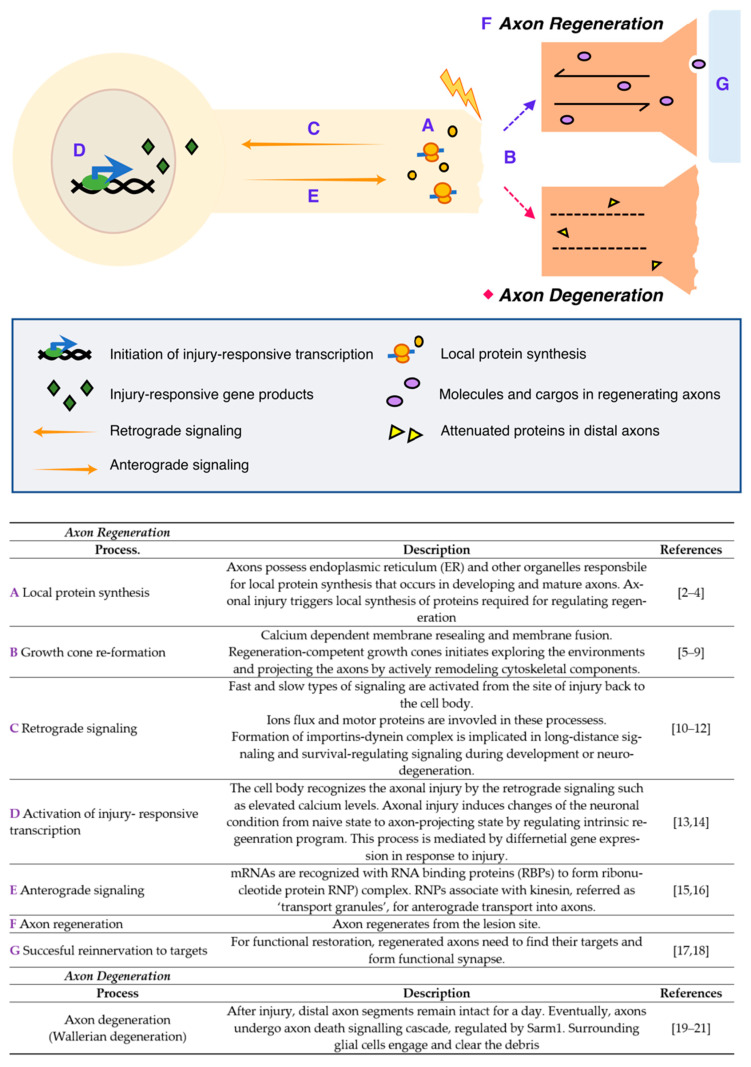
Injury responses in the peripheral nervous system (PNS) [2,3,4,5,6,7,8,9,10,11,12,13,14,15,16,17,18,19,20,21].

Neurons are specialized cells with long and thin projections that are essential for neuronal functions, making connections to their targets throughout the animal body. Although these projections of different neurons generally perform similar functions such as molecular transport and transmitting signals, different types of neurons exhibit strikingly distinctive axonal morphologies. Ramon y Cajal dedicated his life to the illustration and description of neurons and neuroanatomical networks, leading to a better visual understanding—and development of the concepts—of the nervous system [1]. Cajal’s discovery was the decisive evidence for the neuron doctrine, which proposes that the nervous system is a discontinuous entity and is composed of discrete individual cells. In addition, he asked why some nervous tissues in highly evolved animals fail to regenerate after injuries that cause permanent disorders described as a ‘harsh decree’. A number of neurobiologists have discovered key mechanisms of neuronal functions and now we are even uncovering the map of the complete networks from human brains. However, the mechanisms of axon regeneration and degeneration is still under investigation with different models. Neurons are vulnerable to injury due to their projections. Some neurons in the peripheral nervous system (PNS) can regenerate their axons after injury. However, most neurons in the central nervous system (CNS) fail to regenerate, potentially resulting in irreversible neurodegeneration. Therefore, the mechanisms underlying axonal regeneration in the PNS have been extensively investigated to understand regeneration program and to find methods for promoting axon regeneration in CNS. To study axon regeneration, experimentally modeling the whole processes from injury to regeneration is useful as dissecting the complex process governed by multiple regulatory steps (Figure 1).

First, the axons locally synthesize multiple proteins that regulate injury-associated responses after axonal injury, which is mediated by the endoplasmic reticulum (ER) and other organelles required for protein synthesis. Simultaneously, calcium-dependent membrane resealing occurs, and the growth cone is newly constructed by cytoskeletal remodeling. Meanwhile, cell bodies recognize and activate the regeneration programs through injury-induced calcium flux and retrograde signaling that subsequently induce a shift from a naïve to a regenerative state in the neurons. Injury-responsive transcripts are produced and some of them, known as pro-regenerative transcripts or transcripts of regeneration-associated genes (RAG), bind to RNA-binding proteins (RBPs) to form ribonucleotide protein (RNP) complexes; these complexes are known to associate with kinesin and transport to the axons anterogradely. All these processes are coordinately regulated and required for successful axon regeneration as well as axonal reinnervation to the original targets, thereby resulting in the formation of functional synapses and restoring neuronal function. In contrast with regeneration, neurons also show axonal degeneration, an active process of axonal self-destruction. This is known as Wallerian degeneration [22,23,24], a phenomenon that is a characteristic feature that is only seen from neuronal cells. The distal parts of the nerve, containing the axons disconnected from their cell bodies, activate the degeneration process, resulting in the degradation of the myelin sheath and engulfment by macrophages. As briefly described here, axonal injury activates multiple biological processes in distinctive spatiotemporal regulatory systems; for example, a growth cone reforms at the tip of the transected axon, causing the regenerating axon to reinnervate its original target. Therefore, experimental models, specifically designed to investigate individual steps in the processes, are required.

Understanding differential regenerative potential from various conditions is essential for developing applications that promote axonal regeneration in the brain and spinal cord. For examples, glial proteins, such as chondroitin sulfate proteoglycan (CSPG), oligodendrocyte-myelin glycoprotein (OMgp), and myelin-associated glycoprotein (MAG), induced in response to CNS tissue injury, are absent in PNS and serve as molecular barriers that inhibit axonal regeneration [25]. This is one of the most understood key factors responsible for differential regeneration from the CNS and the PNS neurons. Interestingly, neurons in the medulla and adjacent spinal cord regenerate their injured axons into the bridges of the transplanted peripheral nerves, indicating the intrinsic regenerative potential of the CNS neurons [26]. In addition, although the PNS axons generally exhibit robust axonal regeneration, diverse factors can impair the process, thereby causing a permanent neurological deficit by either failing to reinnervate or recover the function properly [27]. These findings emphasize that both the extrinsic and intrinsic neuronal determinants should be targets for understanding the molecular mechanisms of axonal regeneration and that it would require manipulation of all the determinants for complete functional recovery following any injury [28].

To develop methods that promote functional recovery in the adult mammalian CNS and PNS, it is critical to understand the molecular mechanisms that trigger a regenerative response and the associated transcriptional regulation and epigenetic changes in injured neurons [27,29,30]. Dorsal root ganglion (DRG) neurons activate the injury-response that directs axons to either regenerate or degenerate after traumatic nerve injury. Shin et al. compared the injury-induced transcriptomic changes, i.e., differentially expressed genes (DEGs), between the regenerating and degenerating nerve segments [31]. The spatiotemporal profiles of the transcriptomes are different, indicating differential regulation between re- and degenerating neurons. However, further research is needed to determine the complexity of the mechanisms that support regeneration—and their regulation—for the development of effective therapies aimed at promoting neuronal repair in the PNS and CNS [32]. 

Different experimental models help us understand the mechanisms underlying axonal regeneration and identify ways to overcome technical and biological limitations. In this review, we collated results from various experimental models to comprehensively analyze the ability of neurons to regenerate in different systems. The recent innovation in the field of axon degeneration includes the identification of SARM1 as the executioner of axonal self-destruction [19,20]. The mechanisms of Wallerian degeneration and the role of nicotinamide mononucleotide adenylyltransferase 2 (NMNAT2) have been extensively dissected and Sterile Alpha and Toll Interleukin Receptor Motif-containing protein 1 (SARM1) was identified as a positive regulator of axon degeneration [20]. Osterloh et al. identified dSarm by utilizing in vivo axon degeneration assay in a loss-of-function screening in *Drosophila* [20]. The functional analysis of SARM1 revealed that SARM1 is an essential factor of the axon degeneration and triggers an irreversible axonal death [19,33]. Similarly, RNAi-based in vivo screening using the *Drosophila* models identified the new key players regulating axon degeneration such as transmembrane protein 184b (TMEM184b) and MORN repeat containing 4 (MORN4) [34,35]. In addition, an image-based large scale screening analysis identified novel functions of glycogen synthase kinase (GSK3) in axonal degeneration [36]. This loss-of-function analysis was performed at a genome-wide scale efficiently by utilizing in vitro axotomy and degeneration assay method that is introduced in this review. These innovative experimental model systems revolutionize the identifications of new players regulating axon regeneration and degeneration.

## 2. In Vitro Axon Injury Model

**Figure 2:**
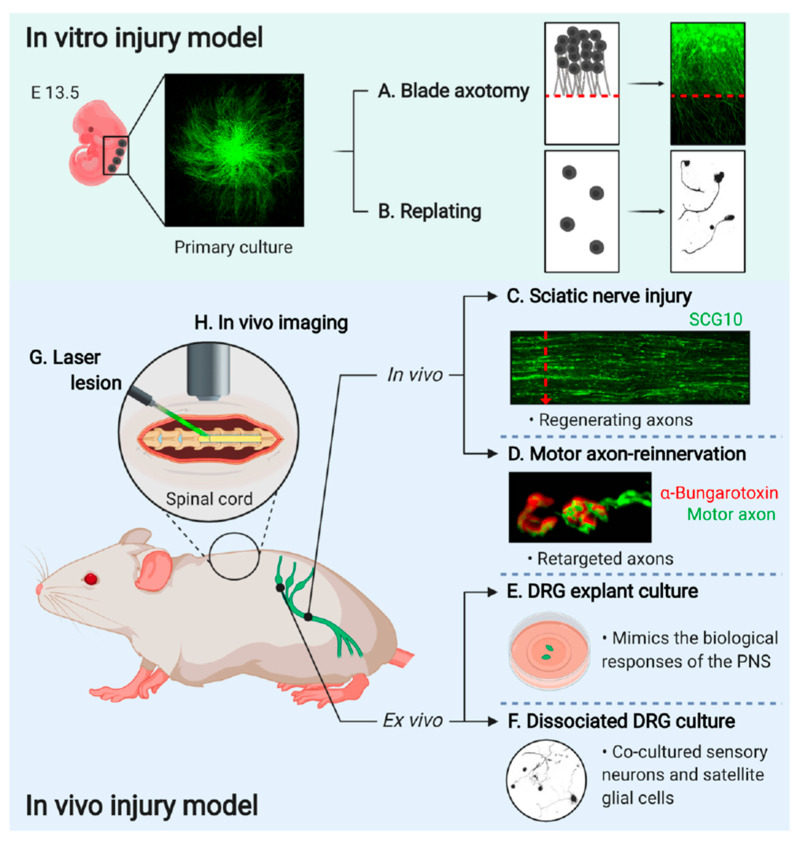
Experimental models to study axon regeneration. (**A**) Primary cultured eDRG neurons are axotomized using a blade and the regenerating axons are investigated [13]. (**B**) eDRG cells are replated to investigate axonal regeneration [37]. (**C**) Sciatic nerve is dissected after three days of injury then immunostained with anti-SCG10 antibody to identify the regenerating axons [38]. (**D**) EHL muscle is observed for the target reinnervation [17]. (**E**) Adult DRGs are trypsinized and dissociated for single-cell culture [14]. (**F**) Adult DRGs are dissected and explanted to investigate the mechanistic pathway more easily [39]. (**G**) A laser lesion to the brain and spinal cord for a better understanding of the dynamics of single axons [40]. (**H**) In vivo imaging is performed by implantation of the spinal window in the spinal cord [41]. (Images created with BioRender.com)

Primary cultured DRG neurons, dissected from embryonic or adult mice, are used to establish a model system for the in vitro regeneration assays and to monitor the axonal outgrowth efficiency with the manipulation of gene expression by viral vector-mediated gene delivery systems. In vitro injury can be induced by two different types of injury; axotomy (Figure 2A) or detaching and replating methods (Figure 2B).

### 2.1. Blade Axotomy

Primary cultured embryonic DRG neurons are prepared using the spot-culture method (Figure 2A). Seven days post-spot-culture, axonal injury is induced by axotomy using a surgical blade (Fine Science Tools, 10035-10), under a stereomicroscope. Axotomy terminates the delivery of all soma-derived cargoes to the distal axons, and therefore axons under the axotomy line undergo self-destructive degeneration. However, the axons that are still connected to the cell body initiates axon regeneration. These regenerating axons are quantified by counting the number of axons that cross the axotomy line within 24 to 40 h after axotomy or utilizing immunofluorescence intensities acquired by particular marker proteins such as βIII tubulin and superior cervical ganglion 10 (SCG10) [13]. Regenerating axons can be labeled with a growth-associated neuronal protein, SCG10, highly expressed in developing and regenerating axons [42,43]. Therefore, the cultures are immunostained with anti-SCG10 and anti-βIII tubulin antibodies, and regeneration indexes are calculated based on the fluorescence intensity of a square area 100 μm distal to the axotomy line and normalized to the same area 100 μm proximal to the axotomy line [13]. This assay enables to monitor the neuronal injury responses directly from the axons and the cell bodies. 

### 2.2. Replating Assay

A replating assay can be used to monitor both axon reformation and regeneration in dispersed single neurons (Figure 2B). The dissociation of the DRGs for primary culture serves as the conditioning lesion. Replated cultured neurons mimic the pre-conditioning effect and exhibit improved neurites [44,45]. Primary cultured embryonic DRG neurons are trypsinized, dissociated into single-cell bodies, and transferred to new culture plates coated with poly-D-lysine/laminin. The replated neurons are incubated overnight at 37 °C in the presence of 5% CO_2_. Axonal regeneration is calculated as a measure of increasing neurite lengths from the cell body [46]. The in vitro axon injury model serves as a convenient system for evaluating the role played by specific genes in axonal regeneration under various injury conditions, providing a supportive evidence as an in vivo validation. In addition, these methods allow monitoring neuronal response without potential effects from glial cells, which is dangerous for leading to misunderstanding the true processes that naturally occur in vivo in neural tissues. Therefore, it is strongly required to remember that these experimental settings have limitations of the artifacts, which is not fully simulated in the in vivo condition.

## 3. In Vivo Axon Injury Model

Genetically modified animals serve as useful models for validating the targets identified based on the in vitro experiments and understanding proof-of-principle axon regeneration after spinal cord injury (Figure 2C,D,G,H) [47]. Diverse options are not available for studying genetics in peripheral sensory neurons, especially in the context of in vivo regeneration. The Advillin-Cre driver mouse line contains Cre recombinase under the regulation of the sensory neuron-specific Advillin promoter. This mouse line is a powerful tool for the targeted expression of genes of interest in sensory neurons, such as DRG neurons [48]. Additionally, the Hb9-Cre mouse is an established model for motor and interneuron-specific gene expression in the spinal cord [49,50]. As it can be used with Cre-dependent recombination to target genes from excitatory interneurons, the Hb9-Cre mouse line efficiently expresses specific genes in postmitotic motor neurons [51].

### 3.1. Sciatic Nerve Injury Model

The sciatic nerve injury model is useful for testing peripheral axon regeneration given the following: (a) its anatomical position is easily accessible and (b) it is structurally long and thick. The sciatic nerves were dissected three days after crush injury and immunostained with SCG10 to selectively label the regenerating axons (Figure 2C). The fluorescent intensity of SCG10 was measured at regular intervals of 100 μm starting from the crush site up to the distal part of the nerve. The intensity is normalized to the intensity at the crush site and presented as a regeneration index [38,52].

The sciatic nerve injury model can be used to perform additional experimental methods. In a previous study, it was revealed that injury to the peripheral branch of DRG neurons—“conditioning lesion”—prior to the injury to the central branch promotes the regeneration of central axons [53,54]. This indicates that retrograde injury signals travel from the peripheral injury site back to the soma to increase the intrinsic growth capacity of the neuron. Furthermore, DRG neurons—hours after injury to the sciatic nerve—respond by activating pro-regenerative factors in the cell body that in turn induce a pro-regenerative program and accelerate axonal regrowth and mediate the preconditioning effect [55]. To assess this, the sciatic nerve was injured for three days to allow the induction of the pro-regenerative program, and then a second crush was applied slightly proximal to the first crush; this allowed the axons to re-grow for one day [56].

Nerve ligation injures axons and blocks axonal transport so that the transported cargoes accumulate near the knots. Retrograde cargoes accumulate in the proximal segment of the nerve whereas, anterograde cargoes are concentrated in the distal segment, so the ratio of protein present in the proximal/distal segment is a measure of the retrograde transport [57,58]. For double ligation experiments, the sciatic nerve is ligated using silk sutures introduced a few millimeters apart. This method enables to monitor differential transports of injury-responsive molecules with directionalities. In addition, immunohistochemistry or biochemical analysis can be applied with this method to quantitively investigate injury-related differential movements of target molecules.

### 3.2. Motor Axon-Reinnervation Model

The functional recovery of motor axons requires successful regeneration not only at the injury site but also at the endplates of the neuromuscular junctions for reinnervation into the target muscle [59]. The extensor hallucis longus (EHL) muscle is used to observe the reinnervation of the motor axons that regenerate through the sciatic nerves in a neuromuscular junction (Figure 2D). The EHL muscles are dissected two weeks following sciatic nerve injury to assess the reinnervation efficiency or speed of growth of motor axons in the sciatic nerve into a neuromuscular junction. Paraformaldehyde-fixed EHL muscles are stained with fluorophore-conjugated α-bungarotoxin to visualize the endplates of neuromuscular junctions. Whole-mounted or flat-mounted samples are microscopically observed to assess the extent of motor axon reinnervation [56].

### 3.3. Spinal Cord Injury Model

Most studies on regeneration in the CNS used retinal ganglion cells of the optic nerve or the spinal cord as the model system [60,61,62]. Relevant models used for clinical translation are contusion and hemisection injury, and these recapitulate of the pathology seen following spinal cord injuries in humans. After traumatic CNS injury, a variety of cell types, including macrophages and astrocytes, invade the injury site. These cells produce fibrotic and astrocytic scar secreting inhibitory factors that define the lesion site and serve as a physical barrier for regenerating axons [63].

Performing a laser lesion causes minimal scarring in the brain and spinal cord of mammals [40,64] (Figure 2G). When lesions are generated in axons using a highly localized laser, the extrinsic injury response is minimized [40]. Thus, the intrinsic response can be studied in more detail with the laser axotomy which can be studied for the dynamics of single axons [40]. Depending on the objective used, the power of the laser and the depth of the axon in the spinal cord determines successful lesioning [41].

### 3.4. In Vivo Imaging

Imaging strategies have been developed to visualize axonal regeneration in the spinal cord; these strategies allow us to investigate simple axonal dynamics after injury and axonal outgrowth after pre- and post-conditioning, retraction bulb formation, and degeneration [5,40,65]. Because wide-field microscopy does not produce images with high spatiotemporal resolution, two-photon imaging is used [41]. Two-photon imaging enables lesion development in single axons with laser pulse [40,66], exhibits deeper tissue penetration, and induces lower photo-toxicity, thereby improving the conditions required for in vivo imaging.

In vivo dynamics in the spinal cord can be visualized acutely or chronically (Figure 2H). For acute visualization, the spinal cord is stabilized with fixed holders on printed 3D microscope inserts [41]. The surgical procedure is performed precisely on a mouse placed under a two-photon microscope, all the while maintaining the physiological conditions [41]. Chronic imaging is performed at different time points after injury, making it stressful for the mouse due to the need for repetitive surgical interventions. Therefore, the spinal window has been developed to perform chronic imaging [67,68,69,70]. The implantation of the spinal windows in the spinal cord enables the imaging of the same axons for up to one year without any damage to the spinal cord [41]. Furthermore, the choice of imaging might depend on the chosen injury model.

## 4. Ex Vivo Injury Model

DRGs extend a single axon with two branches, one goes to the peripheral nerve and the other enters the spinal cord. After the nerve injury, the peripheral branch regenerates, while the central branch does not. Therefore, mouse DRG neurons are a valuable model for studying axon regeneration. There are two methods to culture DRG neurons to better understand neuronal functions in various experiments (Figure 2E,F).

### 4.1. DRG Explant Culture

Explant cultures of adult mouse DRG are used as an organotypic ex vivo injury model (Figure 2E). Once DRGs are dissected, they are plated in a culture dish pre-coated with a gelatinous protein mixture for incubation. DRGs are then gently covered with the culture medium to maintain the explants under culture conditions. The DRG explant culture is more suitable than the single-cell culture models, as it mimics the biological responses associated with the physiological and pathological conditions of the PNS [71]. The explant culture allows for the ex vivo transfer of an entire neuronal network and can be maintained for several days. Therefore, the DRG explant system offers sufficient flexibility to study various events related to biological, physiological, and pathological conditions in a cost-effective manner [71].

### 4.2. Dissociated DRG Neuron Culture

Culture-dissociated adult DRGs serve as another ex vivo model (Figure 2F). Primary sensory neurons and satellite glial cells can be co-cultured—from dissociated DRGs—to investigate the neuronal-glial interaction, neuritogenesis, the interaction of the axonal cone with the extracellular microenvironment, and neuronal metabolism [71]. The details of adult DRG culture have been previously described [72]. Mouse L4 and L5 DRGs are collected and incubated in DMEM supplemented with Liberase Blendzyme 3 (Roche), DNase I (Sigma), and bovine serum albumin for 15 min at 37 °C. The DRGs are then incubated with trypsin-EDTA for 15 min at 37 °C, followed by trituration. Dissociated cells are plated on culture dishes [56].

Both the DRG explants and dissociated neuronal cultures can be virally transduced through treatment with virus-containing medium and subsequent incubation at 37 °C.

## 5. In Vivo Gene Delivery

### 5.1. AAV-Mediated In Vivo Gene Delivery

**Figure 3:**
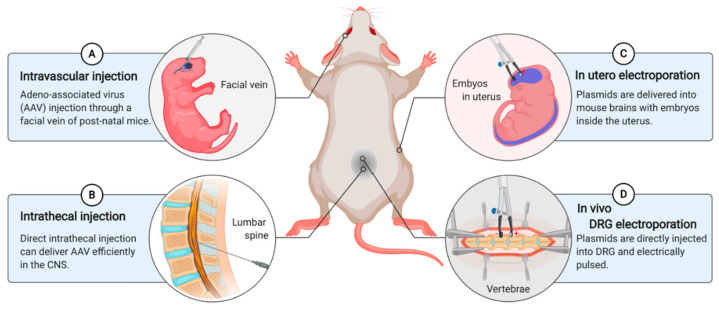
In vivo gene delivery methods to effectively manipulate the target genes. (**A**) AAV-mediated gene delivery enables efficient gene expression in various tissues. AAV-injected mice are suitable for regeneration-related experiments generally after 4 weeks of husbandry. (**B**) The direct intrathecal injection is performed on adult mice which can manipulate the genes in the CNS. Contrast to the intravascular injection, injected mice can be used after 2 weeks post-injection. (**C**) With the electric pulse, plasmids or RNA oligos are introduced into mouse brains at embryonic days 12-17 with the embryos inside the uterus. (**D**) After the microinjection of plasmids or RNA oligos into the adult DRG, electric pulses are delivered to the target DRG. (Images created with BioRender.com)

Genetically modified mice have served as useful in vivo models for performing loss-of-function and gain-of-function analyses for candidate proteins based on axon regeneration in response to various injuries [47]. The development of gene delivery systems is essential to efficiently manipulate genes to analyze the effects of the target gene on the in vivo regenerative capacity. One way to mediate gene delivery is to inject an adeno-associated virus (AAV) through a facial vein into P1 mice (Figure 3A). Certain serotypes of AAV enable effective targeting to the PNS and peripheral organs through intravascular injection in postnatal mice. Following AAV injection, transduction to the dorsal root ganglia, sciatic nerve, liver, heart, skeletal muscle, lung, and myenteric plexus of the gut has been observed [73]. Another way to manipulate the genes by delivering AAV is through direct intrathecal injection (Figure 3B). With intrathecal injection, gene delivery can be mediated safely, noninvasively, and efficiently in the CNS. Therefore, the intrathecal injection strategy can be utilized in therapeutic trials in mouse models to study CNS diseases [74,75]. AAV-mediated gene delivery is useful when specific types of cells in the tissues are targeted for genetic manipulation because different serotypes of AAV can introduce the gene-of-interest efficiently only into specific types of cells.

### 5.2. In Utero Electroporation

In addition to AAV-injection‒mediated gene delivery, in vivo gene delivery can also be achieved through electroporation. Electroporation uses an electric pulse to permeabilize the cell membrane and deliver plasmids or RNA oligos into the cell, and recently into mouse embryos or adult mice [76]. In utero electroporation (IUE) is a method for the effective in vivo manipulation of target genes (Figure 3C). IUE introduces plasmids into mouse brains at embryonic days 12–17 without the removal of embryos from the uterus [77]. IUE is a simple yet powerful tool to efficiently deliver genes to different brain locations. Gene expression persists for a long time, and co-electroporation of fluorescent reporter genes enables the visualization of targeted cells. This technique has the limitation of targeting sporadic brain sections. However, Maschio et al. developed a new technique that allows easy access and highly reliable bilateral transfection at the target brain locations through the use of multiple electrodes [78].

### 5.3. In Vivo DRG Electroporation

Genetic manipulation in adult neurons is a useful strategy for studying genes, which play an important role in all developmental stages because the traditional knockout approach usually results in early embryonic lethality. The manipulation of the genes of interest in the adult stage can ameliorate developmental defects. Therefore, in vivo DRG electroporation was developed for modulating the expression of multiple genes simultaneously to provide a potential tool for dissecting the pathways that regulate axonal regeneration in mammals after either peripheral nerve or spinal cord injuries [79] (Figure 3D). Although in vivo electroporation has a low transfection efficiency compared to that of viral vectors, it has various advantages. For instance, it can be used in almost all tissues and cells. Additionally, either plasmids or small RNA oligos can be injected into the target tissue directly and electrically pulsed, making the procedure less labor-intensive and time-consuming. Moreover, multiple plasmids and RNA oligos can be transfected simultaneously using a single round of electroporation [80].

These strategies allow the study of sensory neurons and axonal regeneration in experiments investigating both gain- and loss-of-function paradigms.

## 6. Recent Innovations Using Experimental Systems

The experimental models presented in this review provide a better understanding of the mechanisms underlying neuronal regeneration and degeneration.

### 6.1. SARM1 and NMNAT2

Wallerian axon degeneration is a form of programmed subcellular death that promotes axonal breakdown after injury [24]. Wallerian degeneration slow (Wlds) mice were identified in the 1980s; these mice display exceptionally delayed in vivo axonal degeneration [81,82,83,84]. The delay of degeneration in Wlds was highly conserved in mice, rats, flies, zebrafish, and primary human neuronal cultures [83,85,86,87,88]. The slow Wallerian degeneration phenotype is attributed to the activity of nicotinamide mononucleotide adenylyltransferase (NMNAT) [89,90,91]. The three isozymes display unique subcellular localization, with NMNAT1 being expressed in the nucleus, and NMNAT2 and NMNAT3 being expressed in the cytoplasm and mitochondria. The reduction in the expression of NMNAT2 post-injury causes NAD+ levels to fall, thereby increasing the NMN/NAD+ ratio [92]. However, Wlds neurons maintain NAD+ levels post-injury [92,93]. Moreover, the loss of NMNAT2 triggers neurite degeneration in neuronal cultures [92]. dSARM, the Drosophila ortholog of Sterile Alpha and Toll Interleukin Receptor Motif-containing protein 1 (SARM1), was initially discovered through a series of amino acid sequence similarity comparisons and is highly conserved in mice, *Caenorhabditis elegans*, and humans [94]. SARM1 was identified as an essential component of the axonal degeneration mechanism through a large-scale genetic screen in Drosophila and a genome-wide RNAi screen in primary mouse neurons [19,20]. The knockdown of SARM1 prevents neuronal degeneration in response to an injury that normally results in neurodegeneration. SARM1 is thought to be activated in response to the lowered levels of NMNAT2, leading to NAD+ depletion and NMN accumulation [19,20,22,33,95,96]. Recently, a novel in vivo gene therapy targeting SARM1 was developed to block axonal degeneration [96].

### 6.2. DLK

Dual leucine zipper kinase (DLK), a mitogen-activated protein kinase, activates c-Jun N-terminal kinases (JNK) and p38 MAPK [56,97,98,99,100]. DLK has an evolutionarily conserved role in regulating neuronal responses to injury [101,102,103,104,105,106,107,108]. It is essential for neural development [97,109] and is involved in injury responses such as axon degeneration and neuronal apoptosis [101,102,103,110]. However, recent studies in *C. elegans*, Drosophila, and mice have demonstrated that DLK is also required for the regenerative response following axotomy [102,104,110]. The expression of DLK protein—present in axons—increases in response to axonal injury [102,111] and is known to promote retrograde injury signaling and axonal regeneration after PNS injury in mammals [56]. Loss of DLK protects the distal axons from Wallerian degeneration [103,105,112] and blocks the injury-induced retrograde JNK signaling with the scaffolding protein JNK-interacting protein 3 (JIP3) [56,58,101]. Consistent with this, the genetic deletion of JNK2/3 is sufficient to protect neurons from degeneration in a range of CNS injury models [113,114,115], although the role of DLK remains unknown. DLK is also an essential molecule for the injury-dependent activation of the transcription factors cJun and STAT3 [56]. The ability of DLK to promote axonal growth and regeneration is in contrast with its role in inducing apoptosis and axon degeneration [105]. Despite substantial progress, our understanding of the mechanism of DLK in response to a multitude of injuries remains limited. The mechanism underlying the difference between the apoptotic and regenerative phenotypes is unclear; however, it could reflect distinct pathways downstream of DLK. Recently, it has been reported that DLK broadly regulates injury-induced transcriptional changes [106]. Moreover, DEG analysis revealed that DLK regulates injury-responsive genes [107]. Additionally, a comparative analysis showed that DLK is required in a retrograde signaling pathway that regulates a regeneration program partially shared between PNS and CNS injury models.

The critical role played by DLK in regeneration in response to neuronal injury suggests that pharmacological inhibition of this kinase may serve as a potential therapeutic strategy for chronic neurodegenerative diseases [108]. This has resulted in the development of a DLK inhibitor, GDC-0134 (RG6000), which has progressed to a phase 1 clinical trial for amyotrophic lateral sclerosis (ALS) [116]. Although there are no other significant reports, DLK inhibitors are promising due to the strong rationale supporting the role of DLK in neurodegeneration. In addition to the development of pharmacological drugs, further studies should also aim to understand the mechanism underlying the spatial and temporal activation of DLK in response to numerous stresses.

## 7. Conclusions and Perspectives

### 7.1. The Advantages of Experimenting with Rodent Models

Rodent models are used in research to mimic human physiology and gene functions to advance our understanding of human diseases. They have been proven valuable for the development of novel drugs for many human conditions. Transgenic mice serve as very useful models for examining the role of specific genes in axonal regeneration after adult injury [62]. Mouse models are important for identifying genes, mechanisms, and pathways that underlie human neurological diseases and are also ideal for testing new therapeutic approaches [117]. New methodologies have increased the speed and accuracy with which new mouse models can be generated, and technological advances have resulted in the development of improved tools to analyze the results obtained using these models. Although rats and mice share many important features, several differences affect the choice of model in neuroscience studies [118]. More studies have been performed in mice than in rats owing to the availability of well-established techniques for genetic disruption. However, the development of tools to alter the rat genome would minimize the differences seen with respect to the use of the two organisms.

### 7.2. Limitations

The use of mouse models to study human biology was based on the genetic and physiological similarities between the two species [119]. Despite the phylogenetic relatedness between humans and mice, there are limitations with respect to the use of mouse models for attaining a comprehensive understanding of human biology due to established differences in size, metabolic rate, life history, microbiomes, and pathogens [119]. Rodent models are also less reliable, owing to the differences in the genetic networks between the two species. When employing mice in biomedical research, one needs to account for the similarities as well as the evolved differences between mice and humans. To ensure that the findings from animal model systems reflect the injury responses in human neurons, it is important to determine whether the epigenomic and transcriptional pathways identified in the animal model also function similarly in humans [27]. Most epigenetic modifications are conserved from mice to humans in the developing retina [120]. However, whether the epigenetic injury responses are conserved between mice and humans remains unknown. One alternative to overcome the limitations of rodent models is using induced pluripotent stem cells (iPSCs), which differentiate into neuronal stem cells upon being injected into the mouse brain [121]. By combining this model with the existing genetic models and reporter mice, it is possible to create a powerful system for analyzing the pathogenesis of neurological disorders.

The establishment of specific standards for disease modeling in mice will improve the reproducibility, reliability, and clinical translation of the findings; this will ensure that the experiments are performed at the highest standard possible within the necessary ethical and regulatory framework. Even though the debate about utilizing the rodent model will continue, it cannot be denied that the information generated upon using rodent models has had a profound impact on human health and will continue to do so [122].

## Abbreviations

**Abbreviation**	**Meaning**
PNS	peripheral nervous system
CNS	central nervous system
RNP	ribonucleotide protein
RBP	RNA binding protein
CSPG	chondroitin sulfate proteoglycan
OMpg	oligodendrocyte-myelin glycoprotein
MAG	myelin-associated glycoprotein
DRG	dorsal root ganglion
DEG	differentially expressed gene
TMEM184b	Transmembrane protein 184b
MORN4	MORN repeat containing 4
GSK3	Glycogen synthase kinase 3
SCG10	superior cervical ganglion 10
EHL	extensor hallucis longus
AAV	Adeno-associated virus
IUE	in utero electroporation
Wlds	Wallerian degeneration slow
NMNAT	nicotinamide mononucleotide adenylyltransferase
SARM1	Sterile Alpha and Toll Interleukin Receptor Motif-containing protein 1
DLK	dual leucine zipper kinase
JNK	c-Jun N-terminal kinase
JIP3	JNK-interacting protein 3
ALS	Amyotrophic lateral sclerosis
iPSC	induced pluripotent stem cell
